# Modelling the impacts of security on construction delays: A case of Afghanistan

**DOI:** 10.1016/j.heliyon.2024.e32662

**Published:** 2024-06-08

**Authors:** Mohammad Basheer Ahmadzai, Kunhui Ye

**Affiliations:** School of Management Science and Real Estate, Chongqing University, 83# Shabei Street, Shapingba District, Chongqing 400045, China

**Keywords:** Afghanistan, Delay causes, Construction projects, Security

## Abstract

Construction projects in conflict-affected regions face unique challenges, with security issues often serving as significant impediments to progress. This research investigates the multifaceted relationship between security factors and construction delays in Afghanistan, aiming to fill a critical gap in existing literature by offering a comprehensive analysis of the specific challenges encountered within this context. Employing a quantitative methodology and engaging stakeholders directly involved in construction projects, the study delves into the intricacies of security-related delay factors, identifying key contributors and assessing their impacts on project timelines. Through quantitative data analysis, the research reveals that security-related issues such as the presence of local armed individuals, interference from tribal leaders, and other forms of insecurity pose significant threats to construction projects in Afghanistan. These factors are found to have both high severity and frequency, underscoring their pervasive influence on project outcomes. Furthermore, the study highlights the interconnected nature of delay factors, emphasizing the need for a holistic approach to project management that integrates security considerations into broader risk management frameworks. Stakeholder perspectives play a central role in the research, with insights gathered from clients, consultants, contractors, and other key actors providing valuable insights into the diverse range of concerns within the construction industry. Variations in risk perceptions and priorities among different stakeholder groups underscore the importance of tailored interventions that account for the specific needs and challenges faced by each group. Building on these insights, the research offers recommendations for enhancing project resilience and mitigating the impacts of security-related delays in Afghanistan. Recommendations include the development and implementation of robust security protocols, proactive risk management strategies, and improved coordination with local authorities and security forces. Moreover, the study identifies avenues for further research, including comparative analyses across different regions and longitudinal studies tracking project outcomes over time. By deepening our understanding of the impacts of security on construction delays, this research contributes to the development of evidence-based policies and practices aimed at promoting sustainable development in conflict-affected regions and beyond.

## 1 Introduction

Construction project delays are a major concern, impacting project performance and often leading to cost overruns, disputes, and even abandonment [[1], [2], [3], [4]]. While construction is crucial for infrastructure development, construction delays pose a major threat to progress, not just globally but also in developing nations like Afghanistan [5,6]. These delays significantly impact the national economy by extending project timelines, increasing costs, and hindering performance in terms of time, cost, and quality [7,8]. Understanding the specific causes of delays in Afghanistan, which faces unique challenges and consistently ranks highest in construction delays globally is vital to address this critical issue [9]. Successful construction in such environments requires careful consideration of both economic and security factors [[10], [11], [12]]. This study aims to model the impact of security on construction project delays within the specific context of Afghanistan, exploring the interconnectedness of these factors and their broader implications.

Construction projects in Afghanistan often fall short of their planned goals, primarily due to construction delays [13,14]. These delays negatively impact both local and foreign investors, significantly hindering the country's reconstruction efforts [15,16]. For example, the Mes Aynak copper mine ($350 million) and the Hajigak iron ore ($550 million), which along with hydrocarbons and gemstones were expected to contribute $1 billion annually, fell short due to security concerns [17]. The government was unable to reach its $1 billion target income in 2020, and it's unlikely to do so in the near future [18]. Similarly, the Turkmenistan–Afghanistan–Pakistan–India (TAPI) Gas Pipeline project, aiming to transport 33 billion cubic meters (BCM) of natural gas annually from Turkmenistan to Afghanistan, Pakistan, and India, was contracted in 2014 but remains incomplete due to security issues [19]. Insecurity and corruption are among the most prominent challenges encountered during project implementation in Afghanistan [20]. While numerous issues hinder construction projects, insecurity stands out as the most significant barrier to the overall success of development programs [21].

A significant number of construction projects in Afghanistan fail to meet their contract deadlines, hindering the country's development process. This widespread issue of delays is directly linked to security concerns [22]. Projects have been delayed and even relocated due to insecurity in planned regions [23]. For example, security concerns prevented necessary surveys in the Baghlan province [24]. Despite these dangers, the implementation of crucial projects continues to be hampered by delays [25]. Insecurity stands out as one of the most pressing challenges for the construction industry in Afghanistan. It not only caused the abandonment of surveys in Baghlan but also poses a constant threat throughout construction phases, causing delays and anxieties for both international and Afghan workers [26]. This poor security environment significantly challenges stakeholders, often leading to project delays and increased costs [27]. In fact, insecurity is considered a leading cause of construction delays in Afghanistan [28]. These delays are a major problem, severely impacting the country's economy and development. While other factors like corruption and inadequate staffing contribute to delays, insecurity remains the most prominent issue, even driving construction workers away from the industry due to safety concerns [29].

Previous research in developing countries like Bangladesh, India, Indonesia, Nepal, Iraq, Iran, and Pakistan acknowledges insecurity as a factor contributing to construction delays. However, a gap exists in understanding the specific security-related causes and their impact. To address this gap, this study investigates the specific security-related causes of construction delays in Afghanistan through stakeholder perspectives and data analysis. This approach will identify and categorize key security factors, analyze their relative influence on project timelines, and explore their complex interactions. By empowering project stakeholders with valuable insights, the findings aim to facilitate the development of effective strategies for mitigating security risks and improving project efficiency, ultimately contributing to Afghanistan's development goals.

## 2 Literature review

Throughout project implementation, a challenging security environment persisted, hampering technical assistance tasks and hindering collaboration on the ground, ultimately delaying development projects in Afghanistan [30,31]. This hindered execution and supervision of activities, further contributing to delays [32]. Security, in fact, stands as a major cause for schedule and budget overruns in most projects [32]. Afghan security forces face a significant challenge in minimizing civilian casualties during ground engagements, the second-leading cause of fatalities and injuries [33]. This translates to Afghan civilians losing their lives in everyday activities due to weak security, jeopardizing their safety while commuting, attending religious services, or simply being near targeted facilities [34].

Insecurity remains a major obstacle to implementing development programs across Afghanistan [35]. While the Mes Aynak project has advanced despite challenges related to security and the archaeological site [36], it remains an exception. Reconstruction efforts span five key areas: funding, security, governance, economic growth, and social development [37]. Security, specifically during site inspection and planning, directly impacts design and construction, while indirectly affecting contracts, materials, quality assurance, and quality control [38]. The ongoing conflict continues to negatively impact everyday life for many Afghans, who express fear simply visiting a bank, attending a tailoring class, entering a courthouse, or celebrating a wedding [39]. This constant insecurity curbs Afghans' ability to exercise their fundamental rights to economic, social, and cultural freedom [40].

Insecurity poses a major obstacle to development projects across Afghanistan, directly impacting design and construction and indirectly affecting contracts, materials, quality assurance, and quality control [41]. This is evident in the delays faced by the Mes Aynak project, despite challenges related to the archaeological site [42]. While SIGAR reports categorize reconstruction efforts into five areas - funding, security, governance, economic growth, and social development [43] - security consistently emerges as a critical factor. The ongoing conflict significantly disrupts everyday life for Afghans, who feel unsafe visiting essential facilities like banks, tailoring classes, courthouses, and even weddings [44]. This insecurity restricts their ability to exercise their fundamental rights to economic, social, and cultural freedom [45]. Specific examples highlight the detrimental impact of insecurity. SIGAR was unable to inspect the Afghan National Army slaughterhouse project in Pol-i-Charkhi due to security concerns, leading to contract termination [46].

Multiple factors contributed to the failure of Afghan rehabilitation programs, including high levels of risk and insecurity faced by participants, a weak economy with limited job opportunities, and inadequate government expertise for program implementation [47]. Insecurity was particularly problematic, hindering operations of humanitarian organizations in many areas [48]. Insurgent attacks targeted government staff and aid workers, and demanded bribes from NGOs and local figures to allow aid distribution [49]. A stark example is the deadly bombing at a Khowst bazaar checkpoint, highlighting the urgent need for security as a foundation for a stable Afghan government [50]. Security concerns, particularly in southern and southeastern regions of Afghanistan, have significantly delayed projects and driven up costs [51]. Addressing these security challenges is crucial to enable contractors to complete projects on schedule [52]. In construction, a project delay refers to completion occurring later than the planned timeline or contractual schedule [53]. Understanding the causes of these delays is essential for minimizing their impact [54,55]. Delays in construction are a major concern within the industry, as they signify missed deadlines and completion dates [56,57]. For Afghanistan specifically, contractor financing uncertainty adds another layer of complication to delay issues [58]. Notably, the challenging security environment in drug-producing regions is considered a key factor behind declining interdiction efforts across the country [59]. Additionally, other notable causes of delays include municipal permission delays, changes in regulatory requirements, and problems with project financing and payments [60].

Studies in Afghanistan highlight security and corruption as the top culprits for construction delays, followed by issues like contractor qualifications, poor site management, and financing challenges [61,62]. Delays in owner payments and specific bidding methods can further contribute [63]. Insecurity has even halted projects entirely in some instances [64]. Beyond construction, security concerns impact grant programs, underlining the critical need to prioritize staff safety [65]. Access to electricity ranks as the top priority for Afghans after security, highlighting the interconnectedness of these issues [66]. Compounding development efforts are challenges like corruption, poor governance, and lack of infrastructure, hindering the use of the country's mineral resources [67]. Similar issues affect construction projects in other developing nations. Studies in Iraq, Bangladesh, India, Pakistan, and Nepal by Ghanim A. Bekr et al. (2015), Rakibul Hoque et al. (2020), Miguel et al. (2008), Irfan Zafar et al. (2016), and Popular Gentle et al. (2012), respectively, all identify security as a major impediment [[68], [69], [70], [71], [72]]. However, existing research often neglects to delve into the specific security factors at play. This study aims to bridge this gap by carefully examining and analyzing the individual security-related factors causing construction delays, offering a deeper understanding of this critical challenge.

**Fig. 1:**
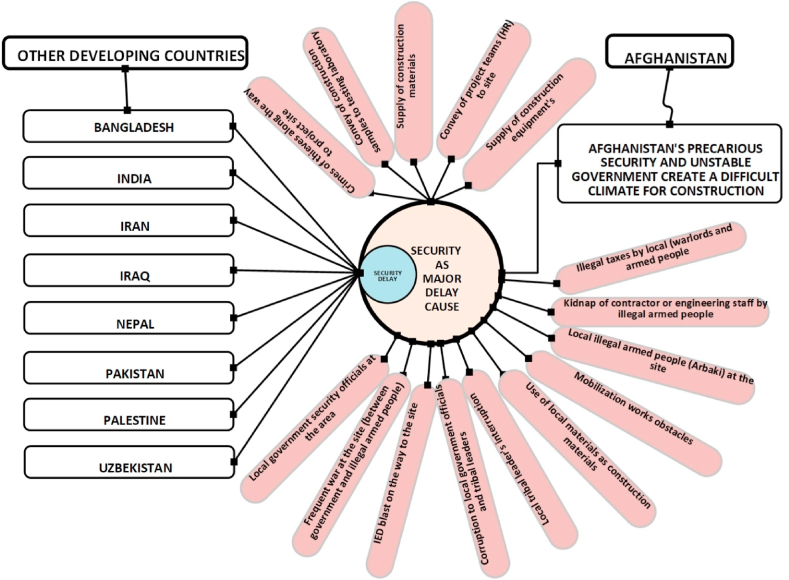
Comparison of security issues affecting construction projects in Afghanistan and other developing countries.

However, Afghanistan's construction industry presents unique challenges in a post-conflict development context, facing an intricate set of challenges unlike those in other developing nations. Numerous studies [[73], [74], [75], [76], [77]] have categorized delay causes into various groups, yet surprisingly, none explicitly analyze security as a distinct category. This omission is especially detrimental for Afghanistan, where security threats directly impact project progress and stand out as the most crucial factor to consider for effective delay reduction [[78], [79], [80]]. While valuable studies have explored construction delays in Afghanistan, they often focus on specific project types [4,17,18,29,and56]], stakeholder perspectives [12,16,42,52,54,and55]], or delay causes [9], [11], [25], [27], [28], [38], limiting their scope and hindering a comprehensive understanding of the issue. This research, however, will delve deeper into the unique context of Afghanistan by systematically examining the causes and consequences of delays across all major types of construction projects, ranking their impacts on the construction projects delay. This research addresses a critical gap in existing literature, providing a deeper understanding of construction delay dynamics in this complex post-conflict environment. Fig. 1 shows the comparison of Security-Related delay in Afghanistan with others developing countries.

## 3 Methodology

This study employed a quantitative methodology, adhering to seven key steps to achieve its objectives. First, a literature review identified the research gap. Second, a pilot survey and interviews were conducted to assess the practicality of data collection and refine the main survey questionnaire. Third, a sample size calculation determined the required number of participants for the main survey. Fourth, data was collected via a questionnaire or main survey. Fifth, data analysis was conducted using statistical methods and the OriginPro software. Sixth, the results and their implications were discussed. Finally, the research concluded and presented recommendations for further studies in the field.

### 3.1 Pilot survey

A pilot survey was conducted with the main aims of improving the survey questionnaire, identifying and addressing potential issues, and optimizing the design and logistics for the successful implementation of the main survey. This involved interviewing Afghan construction experts in the field; 25 participants (7 project managers, 5 lecturers, 8 contractors, and 5 consultants) participated. Through this survey, frequently asked questions were addressed, a survey manual was designed, and two questionnaires were developed for the main survey: one to identify security-related delay causes and the other to rank their impacts on Afghan construction project delays.

### 3.2 Choosing and selecting samples

This research covers various types of construction projects, focusing on 100 Afghan projects (50 urban, 50 rural) to ensure diversity in location, size, and type. Six main project types are included: educational (21), water supply (16), road (21), irrigation (19), military (7), and hospital (16). Stakeholder theory provides a valuable framework for achieving the research aims and objectives. Key stakeholders in Afghan construction projects, as identified by Ghulam Abbas [37], include clients, consultants, contractors, subcontractors, and project managers. Lecturers from relevant academic fields are included as stakeholders due to their involvement in project design and evaluation. To determine the appropriate sample size, we employed the equation proposed by Tanis and Hogg [81]. This resulted in a final sample of 249 participants from various stakeholder groups.

(1) $$n=\frac{m}{1+\left(m-1\right)/N}$$

Where: n = limited sample size, m = unlimited sample size, N = available population, as well as m is calculated by the following equation:

(2) $$m=\left\{Z^{2}*P*\left(1-P\right)\right\}/e^{2}$$

Where: Z is value of confidence used 2.575 for 99 %, 1.96 for 95 %, and 1.645 for 90 % levels of confidence, P is the value of the required population, which is suggested 0.5 by Habibi [88]. So following is the required size of sample for this research.

(2∗) $$m=\left\{{\left(1.96\right)}^{2}*0.5*\left(1-0.5\right)\right\}/{\left(0.05\right)}^{2}=385$$

As the total available stakeholders for the 100 construction projects are 700, then the required numbers of the participants can find with the equation.1 as follow:

(1∗) $$n=\frac{385}{1+\left(385-1\right)/700}=248.6116=249$$

Regarding the above calculation, 249 stakeholders are required for the data collection to represent the total of 700 stakeholders for the research.

### 3.3 Data collection

In Afghanistan, a questionnaire survey was conducted, building upon existing research [23,34,54]. Informed by interviews with Afghan construction experts during a pilot survey, the initial questionnaire concept was refined and resulted in two distinct instruments for quantitative data collection. The first questionnaire aimed to identify security-related delay causes and their causative groups, while the second assessed the impact of each delay cause on construction projects. Of the 275 questionnaires distributed, 250 (50 clients, 49 consultants, 48 contractors, 52 lecturers, and 51 project managers), were returned, yielding a 91 % response rate. Participants utilized two survey formats: online (email, messenger, WhatsApp) and paper-based. Table 1, Table 2, Table 3, Table 4, Table 5 detail participant characteristics.

**Table 1:** Age information.

Description	Client	Consultant	Contractor	Lecturer	project manager	Total
20–30 years old	15	18	13	22	22	90
31–40 years old	17	15	19	12	12	75
41–50 years old	11	11	9	14	14	59
Above 50 years old	7	5	7	4	3	26
Total	50	49	48	52	51	250

**Table 2:** Education.

Description	Client	Consultant	Contractor	Lecturer	project manager	Total
PhD	5	4	1	10	3	23
Master	23	23	12	24	26	108
Bachelor	21	20	22	18	21	102
High school	1	2	13	0	1	17
Total	50	49	48	52	51	250

**Table 3:** Experience.

No	Client	Consultant	Contractor	Lecturer	project manager	Total
Less than three years	4	6	9	5	7	31
Three to six years	21	19	19	11	13	83
Six to ten years	18	17	11	17	17	80
More than ten years	7	7	9	19	14	56
Total	50	49	48	52	51	250

**Table 4:** Occupation.

No	Client	Consultant	Contractor	Lecturer	project manager	Total
Civil engineer	26	21	19	20	17	103
Construction manager	8	13	15	12	16	64
Site Supervisor	9	11	8	13	9	50
Quantity Surveyor	7	4	6	7	9	33
Total	50	49	48	52	51	250

**Table 5:** Regional information.

Description	Client	Consultant	Contractor	Lecturer	project manager	Total
Central Highland	8	5	3	6	6	28
Central Region	4	7	9	8	9	37
Eastern Region	6	4	10	2	7	29
North Eastern Region	7	8	8	3	5	31
Northern Region	6	5	3	7	9	30
South Eastern Region	7	7	5	11	6	36
Southern Region	5	6	6	9	5	31
Western Region	7	7	4	6	4	28
Total	50	49	48	52	51	250

## 4 Data analysis

### 4.1 Ranking the impact of delay causes

The collected data was statistically analyzed to achieve the research objectives. Following the methodology of Fani Antoniou (2021), the impacts of delay causes were ranked using the Risk Priority Number (RPN), which assigns a single numerical value to each delay cause by considering three critical factors: severity (impact of the delay), frequency (likelihood of occurrence), and detection probability (chance of identifying it before it happens) [82,83]. By incorporating these three factors, RPN provides a quantitative basis for prioritization and clear communication of risks, making it a valuable tool for decision-making [84]. High RPN values highlight areas demanding immediate attention, guiding resource allocation and mitigation efforts. While RPN requires accurate data and may involve some subjectivity in assigning values, it remains a valuable tool for construction project management by empowering teams to make informed decisions and minimize the negative impacts of unforeseen disruptions [85,86].

(3) RPN=SIxFIxDI

Where: RPN= Risk priority Number, SI = severity Index, FI = Frequency Index, and DI = Detection probability.

(4) $$S.I=\sum_{i=1}^{5}\left(a_{i}.\text{ni}\right)/5N$$

Severity Index (S·I.): Measures the perceived severity of each delay cause, considering responses from participants. Scores range from 1 (very low) to 5 (very high), n = frequency responses for every cause of delay, and N = response total amount.

(5) $$F.I=\sum_{i=1}^{5}\left(b_{i}.\text{ni}\right)/5N$$

Frequency Index (F·I.): Assesses how often each delay cause typically occurs in projects. Similar to S·I., scores range from 1 (not happened) to 5 (always happened).

(6) R.V=S.IxF.I

Risk Value (R.V.): Combines S.I. and F.I. to provide a holistic view of a delay cause's overall significance. It reflects both its potential impact and how often it occurs. R.V. scores range from 0 to 1, with values of 0.5 or higher indicating the most critical causes [87,88].

(7) $$D.I=\sum_{i=1}^{5}\left(c_{i}.\text{ni}\right)/5N$$

Detectability Index (D.I.): Estimates the likelihood of identifying each delay cause before it significantly affects the project. Scores range from 1 (very low detectability) to 5 (very high detectability). Higher scores indicate a better chance of early detection, which can mitigate its impact.

The RPN analysis (Table 6) revealed valuable insights into the relative impact of delay causes. Security concerns emerged as prominent factors, with “Local illegal armed people at the site” ranking highest (RPN = 0.2184). Additionally, corruption among local officials and tribal leaders also presented a significant challenge (RPN = 0.2466). Interestingly, logistical issues such as “Supply of construction equipment's" (RPN = 0.2231) and “Convey of project teams (HR) to site” (RPN = 0.2084) also featured prominently.

**Table 6:** Ranking the impact of delay causes.

No	Delay causes	S·I	R	F·I	R	R. V	R	D.I	R	R.P·N	R
1	Local government security officials at the area	0.5680	15	0.5071	15	0.2880	15	0.6141	1	0.1769	12
2	War at the site	0.6922	2	0.6312	5	0.4369	4	0.5829	7	0.2547	3
3	IED blast on the way to the site	0.5770	13	0.5160	14	0.2977	14	0.5911	6	0.1760	14
4	Corruption to local government officials and tribal leaders	0.6691	7	0.6082	9	0.4070	8	0.6059	2	0.2466	4
5	Local tribal leader's interruption	0.6907	3	0.6297	6	0.4350	5	0.6015	4	0.2616	2
6	Use of local materials as construction materials	0.5903	12	0.5294	13	0.3125	13	0.6045	3	0.1889	11
7	Mobilization works obstacles	0.6818	5	0.6208	7	0.4233	7	0.5063	11	0.2143	8
8	Local illegal armed people at the site	0.7004	1	0.6394	4	0.4478	2	0.4877	14	0.2184	6
9	Kidnap of contractor or engineering staff by illegal armed people	0.6439	9	0.6171	8	0.3973	9	0.4818	15	0.1914	10
10	Illegal taxes by local (warlords and armed people)	0.6773	6	0.6506	2	0.4406	3	0.5955	5	0.2624	1
11	Supply of construction equipment's	0.6877	4	0.6610	1	0.4546	1	0.4907	13	0.2231	5
12	Convey of project teams (HR) to site	0.6126	10	0.5859	10	0.3589	10	0.5807	8	0.2084	9
13	Supply of construction materials	0.6669	8	0.6401	3	0.4269	6	0.5041	12	0.2152	7
14	Convey of construction samples to testing laboratory	0.5926	11	0.5658	11	0.3353	11	0.5264	10	0.1765	13
15	Crimes of thieves along the way to project site	0.5740	14	0.5472	12	0.3141	12	0.5576	9	0.1751	15

Note: SI = Severity Index, FI = Frequency Index, RV = Risk Value, DI = Detection probability, RPN = Risk Priority Number, R = Rink.

### 4.2 Assessments of delay causes

**Fig. 2:**
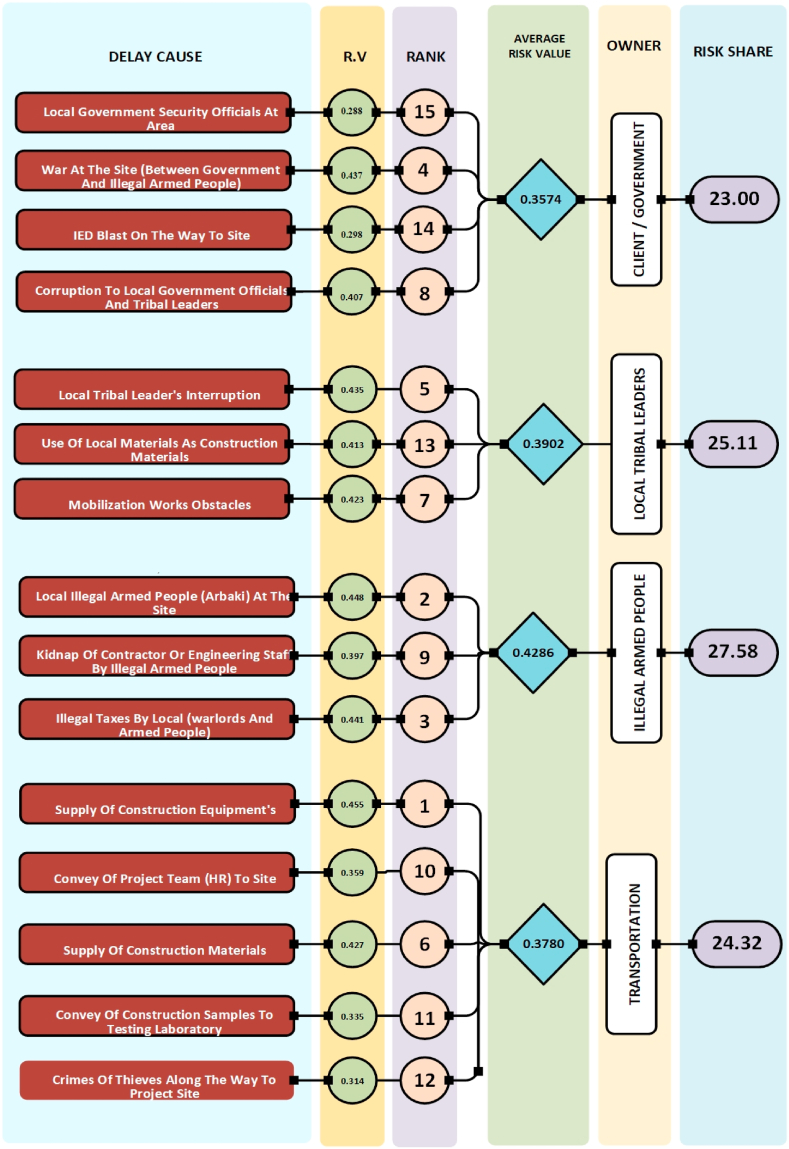
Analysis of delay cause based on Risk Value (RV).

**Fig. 3:**
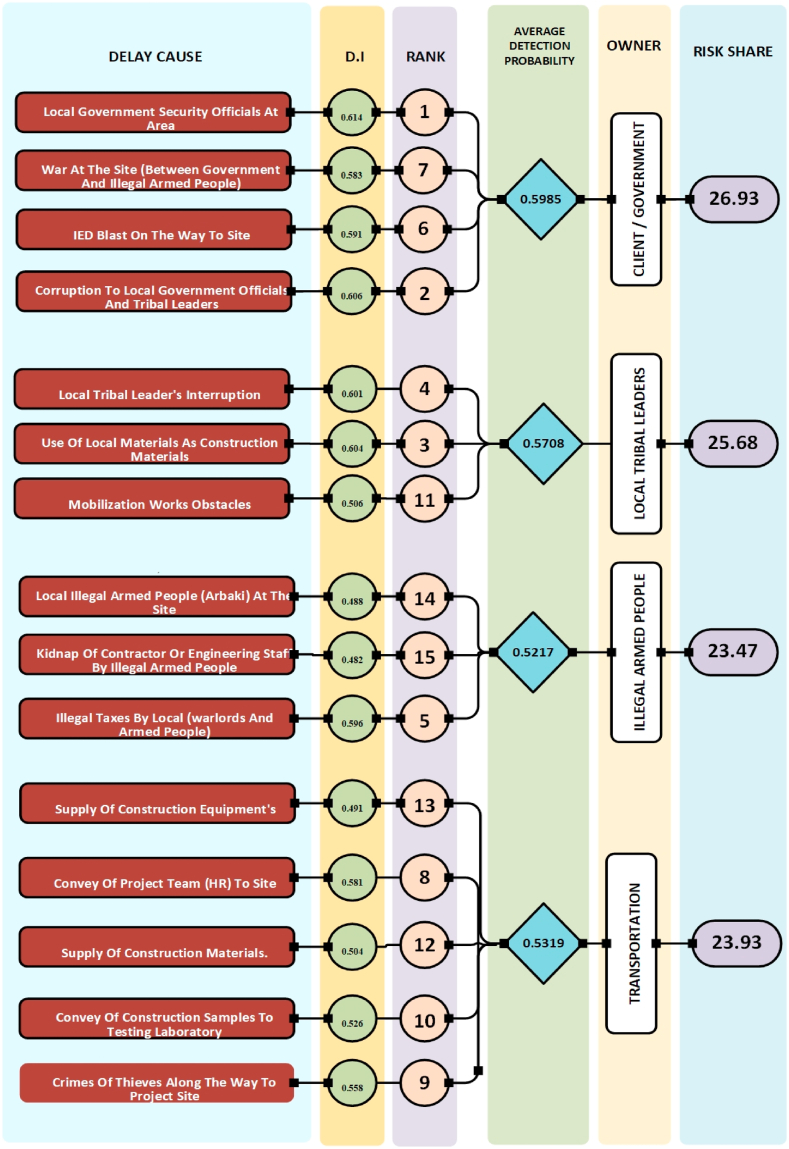
Analysis of delay cause based on Detection Probability (DI).

**Fig. 4:**
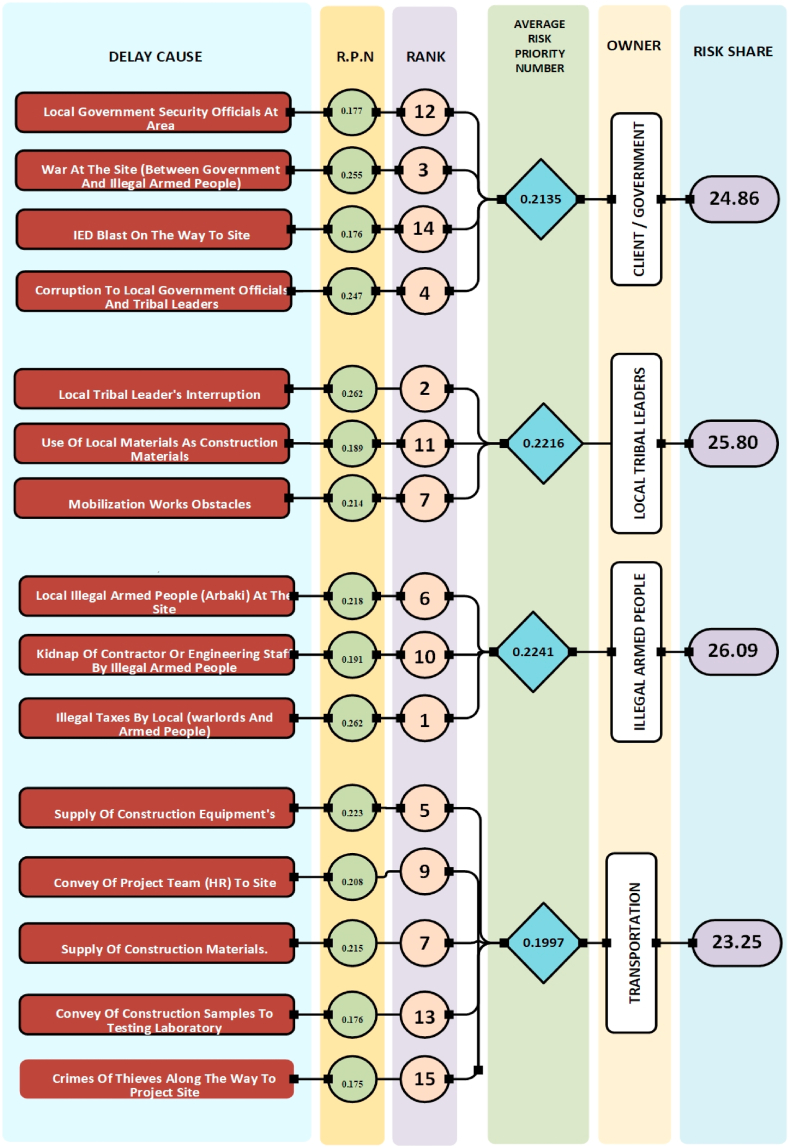
Analysis of delay cause based on Risk Priority Number (RPN).

This section analyzes the key factors contributing to construction project delays using three crucial indices: Risk Value (RV), Detectability Index (DI), and Risk Priority Number (RPN). Fig. 2, Fig. 3, Fig. 4 visually represent the ranking of each delay cause's impact, along with the average values for each main causative group and their overall risk contribution.

Analysis of Fig. 2, Fig. 3, Fig. 4 reveals differing risk shares among stakeholders. Based on Risk Value (RV), Fig. 2 shows the Client group bearing the lowest risk (23 %), followed by Transportation (24.32 %), Local Tribal Leaders (25.11 %), and Illegal Armed People (27.58 %). Conversely, Fig. 3's Detection Probability (DI) data depicts a slightly different picture, with Clients holding the highest detection likelihood (26.93 %), followed by Transportation (23.93 %), Local Tribal Leaders (25.68 %), and Illegal Armed People (23.47 %). Finally, Fig. 4's Risk Priority Number (RPN) analysis places Client risk share in the middle (24.86 %), with Local Tribal Leaders (25.80 %) and Illegal Armed People (26.09 %) holding slightly higher shares, while Transportation occupies the lowest position (23.25 %).

### 4.3 The correlation between importance and frequency of occurrence

To assess the relationship between the importance and frequency of delay causes for each group, Spearman's rank correlation coefficient was employed. Correlation measures the degree of association between two variables [89]. This formula allows us to identify positive or negative correlations and their strength, ranging from −1 (perfect negative) to +1 (perfect positive). A value of 0 indicates no correlation [90]. The formula used is:

(8) $$R=1-\left[\left\{6\sum_{n=1}^{n}d^{2}\right\}/\;\left(n^{3}-n\right)\right]$$

Where R is the Spearman's rank correlation coefficient between importance and frequency of occurrence, d is the difference between the ranks assigned to each cause, and n represents the number of pairs of ranks.

Table 7, Table 8, Table 9, Table 10 present the correlation values for all four main delay groups (government, local tribal leaders, illegal armed groups, and way-related). The analysis reveals a positive relationship between importance and frequency for all groups, but the strength of correlation varies. The way-related group exhibits the strongest correlation, while the local tribal leaders' group shows the weakest.

**Table 7:** Correlation of the government-related causes.

No	Delay causes	Importance Index	R_1_	Frequency Index	R_2_	D	D^2^	R
1	Local government security officials at the area	0.604	4	0.583	4	0	0	0.775
2	War at the site (Between government and illegal armed people)	0.589	3	0.524	1	2	4	
3	IED blast on the way to site	0.582	2	0.554	3	−1	1	
4	Corruption to local government officials and tribal leaders	0.564	1	0.546	2	−1	1

**Table 8:** Correlation of the local tribal leaders-related causes.

No	Delay causes	Importance Index	R_1_	Frequency Index	R_2_	D	D^2^	R
1	Local tribal leader's interruption	0.577	3	0.536	2	1	1	0.703
2	Use of local materials as construction materials	0.553	2	0.487	1	1	9	
3	Mobilization works obstacles	0.552	1	0.54	3	−1	1

**Table 9:** Correlation of the illegal armed people-related causes.

No	Delay causes	Importance Index	R_1_	Frequency Index	R_2_	D	D^2^	R
1	Local illegal armed people (Arbaki) at the site	0.577	3	0.536	2	1	0	0.898
2	Kidnap of contractor or engineering staff by illegal armed people	0.553	2	0.487	1	1	9	
3	Illegal taxes by local (warlords and armed people)	0.552	1	0.51	2	−1	1

**Table 10:** Correlation of the way-related causes.

No	Delay causes	Importance Index	R_1_	Frequency Index	R_2_	D	D^2^	R
1	Supply of construction equipment's	0.669	5	0.625	5	0	0	0.975
2	Convey of project team (HR) to site	0.647	4	0.608	4	0	0	
3	Supply of construction materials.	0.645	3	0.606	3	0	0	
4	Convey of construction samples to testing laboratory	0.615	2	0.554	1	1	1	
5	Crimes of thieves along the way to project site	0.614	1	0.575	2	−1	1	

### 4.4 Ranking of the causes of delays from the viewpoints of key stakeholders

To evaluate and rank the causes of delay from the perspectives of various participants, Assaf's (2005) statistical techniques were employed. Specifically, the “Important Index (P)" was used, where:

(9) $$P=\sum_{i=1}^{5}a\left(n/N\right)*100/5$$

Here, “a" represents a weighting factor assigned to each response (ranging from 1 for very low to 5 for very high), “n" is the number of respondents indicating that cause, and “N" is the total number of participants. Higher P values (above 0.11) signify the most impactful delay causes, while lower values (0.11 and below) indicate the least impactful ones [91].

*Note: VLI = very low influence, LI = low influence, MI = medium influence, HI = high influence, VHI = very high influence, and OI = overall influence.

From the client's perspective, the most impactful delay causes include kidnapping of personnel, local tribal interference, material supply challenges, wartime disruptions, mobilization obstacles, and theft on the project route. They consider illegal taxes, armed groups' presence, IED blasts, local government corruption, sample testing delays, and security personnel involvement to be less significant delay factors.

Consultants identified kidnapping of personnel, material supply issues, local security concerns, wartime disruptions, theft on the project route, and mobilization obstacles as the most impactful delay factors. They considered factors like illegal taxes, tribal interference, armed groups' presence on-site, sample testing delays, IED blasts, local government corruption, equipment supply, and use of local materials to be less significant.

From the contractor's perspective, the most critical delay factors are material supply challenges, personnel kidnapping, local security concerns, theft on the project route, wartime disruptions, and using local materials. They consider less significant delays to be caused by illegal taxes, sample testing, tribal interference, armed groups' presence on-site, mobilization obstacles, IED blasts, equipment supply, and local government corruption.

Lecturers ranked illegal taxes, material supply challenges, personnel kidnapping, theft on the project route, wartime disruptions, sample testing delays, security concerns from local officials, and equipment supply as the most impactful delay factors. They considered corruption, tribal interference, mobilization obstacles, IED blasts, use of local materials, armed groups' presence on-site, and project team travel to be less significant contributors to delays.

Project managers identified kidnapping of personnel, material supply issues, wartime disruptions, using local materials, project team travel, and theft on the project route as the most significant delay factors. They found delays related to equipment supply, illegal taxes, tribal interference, local government corruption, mobilization obstacles, presence of armed groups, IED blasts, security personnel involvement, and sample testing to be less notable.

An analysis of stakeholder perceptions using Assaf's (2005) method revealed varying priorities (Table 11, Table 12, Table 13, Table 14, Table 15). Clients (Table 11) ranked material supply challenges, followed by personnel kidnapping, local security concerns, and wartime disruptions, as the most critical delay factors. Consultants (Table 12) echoed similar concerns but placed a higher emphasis on kidnapping and security issues. Contractors (Table 13) identified material supply and security concerns as top priorities, while lecturers (Table 14) highlighted the negative impact of illegal taxes and material supply constraints. Project managers (Table 15) found material supply, personnel safety, and wartime disruptions to be the most significant delay factors.

**Table 11:** Ranking of the causes of delay, from the viewpoints of client.

No	Delay causes	VLI	LI	MI	HI	VHI	OI(P)
1	Local government security officials at the area	0.11	0.12	0.12	0.09	0.06	0.110
2	War at the site (Between government and illegal armed people)	0.1	0.1	0.09	0.16	0.05	0.117
3	IED blast on the way to site	0.12	0.11	0.14	0.08	0.05	0.106
4	Corruption to local government officials and tribal leaders	0.1	0.14	0.13	0.1	0.03	0.106
5	Local tribal leader's interruption	0.03	0.02	0.03	0.22	0.2	0.163
6	Use of local materials as construction materials	0.14	0.08	0.1	0.11	0.07	0.111
7	Mobilization works obstacles	0.09	0.12	0.1	0.13	0.06	0.116
8	Local illegal armed people (Arbaki) at the site	0.11	0.14	0.13	0.07	0.05	0.105
9	Kidnap of contractor or engineering staff by illegal armed people	0.02	0.04	0.03	0.18	0.23	0.165
10	Illegal taxes by local (warlords and armed people)	0.17	0.13	0.11	0.05	0.04	0.093
11	Supply of construction equipment's	0.13	0.07	0.13	0.12	0.05	0.111
12	Convey of project team (HR) to site	0.13	0.11	0.1	0.1	0.06	0.108
13	Supply of construction materials	0.02	0.03	0.04	0.21	0.2	0.163
14	Convey of construction samples to testing laboratory	0.1	0.13	0.12	0.13	0.02	0.107
15	Crimes of thieves along the way to project site	0.1	0.1	0.13	0.1	0.07	0.115

**Table 12:** Ranking of the causes of delay, from the viewpoints of consultant.

No	Delay causes	VLI	LI	MI	HI	VHI	OI(P)
1	Local government security officials at the area	0.08	0.12	0.11	0.11	0.07	0.115
2	War at the site (Between government and illegal armed people)	0.11	0.09	0.09	0.14	0.06	0.114
3	IED blast on the way to site	0.11	0.11	0.15	0.07	0.05	0.105
4	Corruption to local government officials and tribal leaders	0.1	0.15	0.09	0.11	0.04	0.105
5	Local tribal leader's interruption	0.15	0.1	0.1	0.1	0.04	0.100
6	Use of local materials as construction materials	0.12	0.1	0.1	0.09	0.08	0.110
7	Mobilization works obstacles	0.1	0.09	0.13	0.12	0.05	0.112
8	Local illegal armed people (Arbaki) at the site	0.11	0.14	0.12	0.08	0.04	0.102
9	Kidnap of contractor or engineering staff by illegal armed people	0.02	0.04	0.03	0.19	0.21	0.160
10	Illegal taxes by local (warlords and armed people)	0.15	0.15	0.1	0.05	0.04	0.092
11	Supply of construction equipment's	0.07	0.17	0.13	0.07	0.05	0.106
12	Convey of project team (HR) to site	0.1	0.13	0.08	0.11	0.07	0.111
13	Supply of construction materials	0.02	0.03	0.04	0.21	0.19	0.159
14	Convey of construction samples to testing laboratory	0.1	0.13	0.12	0.12	0.02	0.104
15	Crimes of thieves along the way to project site	0.1	0.09	0.13	0.1	0.07	0.114

**Table 13:** Ranking of the causes of delay, from the viewpoints of contractor.

No	Delay causes	VLI	LI	MI	HI	VHI	OI(P)
1	Local government security officials at the area	0.1	0.08	0.12	0.1	0.08	0.114
2	War at the site (Between government and illegal armed people)	0.1	0.1	0.08	0.13	0.07	0.113
3	IED blast on the way to site	0.11	0.11	0.15	0.05	0.06	0.102
4	Corruption to local government officials and tribal leaders	0.1	0.11	0.09	0.12	0.06	0.110
5	Local tribal leader's interruption	0.15	0.1	0.1	0.09	0.04	0.097
6	Use of local materials as construction materials	0.09	0.09	0.12	0.13	0.05	0.112
7	Mobilization works obstacles	0.13	0.11	0.09	0.12	0.03	0.100
8	Local illegal armed people (Arbaki) at the site	0.11	0.14	0.11	0.08	0.04	0.099
9	Kidnap of contractor or engineering staff by illegal armed people	0.05	0.05	0.03	0.24	0.11	0.140
10	Illegal taxes by local (warlords and armed people)	0.16	0.13	0.1	0.05	0.04	0.090
11	Supply of construction equipment's	0.07	0.17	0.12	0.07	0.05	0.104
12	Convey of project team (HR) to site	0.08	0.12	0.13	0.06	0.09	0.112
13	Supply of construction materials	0.07	0.04	0.03	0.13	0.21	0.145
14	Convey of construction samples to testing laboratory	0.14	0.15	0.09	0.05	0.05	0.093
15	Crimes of thieves along the way to project site	0.09	0.09	0.13	0.1	0.07	0.113

**Table 14:** Ranking of the causes of delay, from the viewpoints of lecturer.

No	Delay causes	VLI	LI	MI	HI	VHI	OI(P)
1	Local government security officials at the area	0.11	0.14	0.12	0.1	0.05	0.112
2	War at the site (Between government and illegal armed people)	0.11	0.09	0.11	0.15	0.06	0.122
3	IED blast on the way to site	0.12	0.12	0.16	0.07	0.05	0.110
4	Corruption to local government officials and tribal leaders	0.15	0.11	0.13	0.09	0.04	0.106
5	Local tribal leader's interruption	0.15	0.1	0.12	0.1	0.05	0.109
6	Use of local materials as construction materials	0.14	0.12	0.09	0.13	0.04	0.110
7	Mobilization works obstacles	0.11	0.14	0.15	0.08	0.04	0.109
8	Local illegal armed people (Arbaki) at the site	0.15	0.12	0.08	0.1	0.07	0.110
9	Kidnap of contractor or engineering staff by illegal armed people	0.02	0.07	0.06	0.19	0.18	0.160
10	Illegal taxes by local (warlords and armed people)	0.03	0.02	0.04	0.24	0.19	0.168
11	Supply of construction equipment's	0.13	0.13	0.1	0.1	0.06	0.111
12	Convey of project team (HR) to site	0.14	0.13	0.07	0.13	0.05	0.110
13	Supply of construction materials	0.05	0.02	0.06	0.18	0.21	0.163
14	Convey of construction samples to testing laboratory	0.11	0.1	0.13	0.11	0.07	0.119
15	Crimes of thieves along the way to project site	0.11	0.1	0.1	0.13	0.08	0.122

**Table 15:** Ranking of the causes of delay, from the viewpoints of project manager.

No	Delay causes	VLI	LI	MI	HI	VHI	OI(P)
1	Local government security officials at the area	0.11	0.14	0.11	0.1	0.05	0.110
2	War at the site (Between government and illegal armed people)	0.11	0.09	0.08	0.17	0.06	0.121
3	IED blast on the way to site	0.12	0.12	0.15	0.07	0.05	0.107
4	Corruption to local government officials and tribal leaders	0.13	0.15	0.08	0.11	0.04	0.105
5	Local tribal leader's interruption	0.15	0.11	0.12	0.09	0.04	0.103
6	Use of local materials as construction materials	0.09	0.15	0.1	0.13	0.04	0.113
7	Mobilization works obstacles	0.15	0.1	0.11	0.1	0.05	0.106
8	Local illegal armed people (Arbaki) at the site	0.14	0.12	0.08	0.13	0.04	0.107
9	Kidnap of contractor or engineering staff by illegal armed people	0.05	0.04	0.03	0.19	0.2	0.158
10	Illegal taxes by local (warlords and armed people)	0.17	0.11	0.16	0.03	0.04	0.095
11	Supply of construction equipment's	0.16	0.15	0.11	0.05	0.04	0.095
12	Convey of project team (HR) to site	0.08	0.16	0.14	0.08	0.05	0.111
13	Supply of construction materials	0.03	0.02	0.05	0.19	0.22	0.166
14	Convey of construction samples to testing laboratory	0.17	0.07	0.09	0.1	0.08	0.110
15	Crimes of thieves along the way to project site	0.09	0.16	0.12	0.08	0.06	0.111

## 5 Results and discussion

### 5.1 Result

This study employed various metrics to assess the impact of security on construction delays in Afghanistan. By analyzing Risk Priority Number (RPN), we identified that local armed individuals and tribal leaders pose the greatest threats, followed by war, corruption, and interference from armed groups. These factors exhibit high severity and frequency, emphasizing their significant and recurring impact. However, security isn't the only challenge. Material supply issues, local governance hurdles, and infrastructural deficiencies also significantly contribute to delays, highlighting the multifaceted nature of delay factors in this environment. Stakeholder analysis revealed variations in perceived risk and impact. While clients generally face lower risks, transportation-related issues, local tribal leaders, and illegal armed groups play a significant role for others. Interestingly, different groups prioritize differently, with clients concerned about material supply and tribal interference while consultants emphasize sample testing and security involvement. This diversity underlines the importance of considering stakeholder perspectives when developing mitigation strategies.

The positive correlation between perceived importance and frequency of delays suggests common ground for intervention. However, recognizing the diverse perspectives and varying risk shares among stakeholders is crucial. Targeted mitigation strategies that address both the severity and frequency of delays, while considering the specific concerns of different stakeholder groups, will be most effective in combating the complex challenges plaguing construction projects in Afghanistan and similar contexts. Overall, the research findings provide valuable insights for policymakers, project managers, and other stakeholders involved in Afghan construction projects. By acknowledging the critical role of security-related factors while also recognizing the broader contextual challenges at play, stakeholders can develop more effective strategies to mitigate delays and ensure project success. Moreover, the findings offer valuable lessons for similar contexts, emphasizing the importance of tailored approaches that account for the diverse array of factors influencing construction projects in developing nations grappling with security concerns and infrastructure challenges.

### 5.2 Discussion

This research delves into the intricate relationship between security and construction delays in Afghanistan, uncovering valuable insights for both theory and practice. By employing a quantitative approach and engaging diverse stakeholders, it sheds light on the specific challenges faced in this volatile environment. Firstly, the research pinpoints critical security factors like local armed groups and tribal leaders as major threats, emphasizing the need for targeted interventions. Strategies like enhanced security protocols, community engagement, and collaboration with authorities can mitigate these risks and bolster project resilience. However, a holistic approach is crucial. The study highlights the multifaceted nature of delays, where material supply issues and governance hurdles also play significant roles. Integrating security considerations into broader project planning and risk management becomes essential to tackle these challenges comprehensively.

Furthermore, stakeholder perspectives reveal diverse concerns and priorities. Clients may worry about material supply and tribal interference, while consultants emphasize sample testing and security involvement. Recognizing these unique views allows policymakers and project managers to develop effective strategies that address the specific needs of different groups. Stakeholder engagement and collaboration are hence vital for building a more resilient and sustainable construction sector in Afghanistan. In conclusion, this research offers not only a deeper understanding of security's impact on Afghan construction delays but also valuable insights for developing targeted interventions and policies to enhance project resilience and mitigate risks. Additionally, the lessons learned are likely applicable to other developing nations facing similar security challenges, showcasing the broader relevance and significance of this study in the international development context.

Clients can leverage the identified risk factors (Table 11) to prioritize mitigation strategies, like securing alternative material suppliers or addressing potential tribal interference. This allows for smoother project execution through better resource allocation. Similarly, consultants can utilize the stakeholder-specific risk landscape (Table 11, Table 12, Table 13, Table 14, Table 15) to develop more comprehensive risk management plans tailored to project challenges, including security concerns. This empowers them to advise clients on effective mitigation strategies and inform project risk assessments. Contractors and project managers can also benefit significantly. Contractors can use the insights on security and material supply challenges (Table 13) to proactively address potential delays. This might involve partnering with local security providers or exploring alternative materials. Project managers, empowered by the stakeholder-specific ranking of delay factors (Table 11, Table 12, Table 13, Table 14, Table 15), can make informed decisions regarding project scheduling, resource allocation, and contingency planning. This translates to realistic project schedules that consider potential delays and the establishment of clear contingency plans to address them.

### 5.3 Strength and limitation of the study

Despite efforts to ensure diversity, the study's sample size and regional/temporal focus limit generalizability. Data collection in Afghanistan presented challenges like limited access and security concerns, potentially impacting data and introducing biases. Stakeholder perspectives, while valuable, are inherently subjective and influenced by experiences and interests. Additionally, the quantitative approach, while offering insights, may miss the complexities of issues in conflict zones. Future research should expand the sample, broaden the context, address data limitations, and consider qualitative approaches for a more comprehensive understanding.

## 6 Conclusion and recommendations

### 6.1 Conclusion

This research delved into the intricate network of security-related factors impacting construction delays in Afghanistan, offering valuable insights for both theory and practice. By employing a quantitative approach and engaging diverse stakeholders, it illuminated the specific challenges plaguing projects in this volatile environment. The research identified critical security factors like local armed groups and tribal leaders as major threats, highlighting the urgent need for targeted interventions. These factors, along with war, corruption, and interference from armed groups, were ranked based on their impact using the Risk Priority Number (RPN) methodology, emphasizing their pervasive influence on project timelines. However, a holistic understanding demands acknowledging the multifaceted nature of delays. The study revealed that material supply constraints and governance issues also play significant roles, underscoring the importance of integrating security considerations into broader project management frameworks. Furthermore, stakeholder perspectives were explored to capture the diverse range of concerns and priorities within the Afghan construction industry. While clients prioritized material supply and tribal interference, consultants emphasized sample testing and security involvement. Recognizing these unique views is crucial for developing effective and inclusive strategies that address the specific needs of different groups. Stakeholder engagement and collaboration, therefore, emerge as vital elements in fostering a more resilient and sustainable construction sector in Afghanistan.

The research also investigated the relationship between the importance and frequency of delay causes within each stakeholder group. Spearman's correlation coefficient revealed the strongest association for way-related delays, followed by government-related and illegal armed people-related delays. Interestingly, local tribal leader-related delays exhibited the weakest correlation, suggesting further exploration into their complex dynamics. Despite these variations, all five stakeholder groups concurred on the most impactful delays: kidnapping, war, material supply, and theft. Conversely, they agreed that IED blasts, local armed people, and government corruption were least influential. These findings highlight the shared challenges faced by the construction industry while acknowledging the nuanced perspectives of different stakeholders. Our research not only identifies critical risk factors and delay causes in Afghan construction projects, but also offers valuable insights for policymakers aiming to improve the industry's efficiency and security. By addressing the identified challenges, policymakers can contribute to a more streamlined and stable construction environment. Potential areas for policy intervention include mitigating supply chain disruptions and security concerns (Table 11, Table 12). This could involve streamlining import processes, encouraging local material production, fostering partnerships with reliable suppliers, improving security personnel training, facilitating collaboration with local security forces, and establishing clear risk mitigation guidelines. Additionally, enhancing risk management practices and community engagement (Table 11, Table 12, Table 13, Table 14, Table 15) can equip stakeholders with the tools and strategies to navigate complex challenges and minimize project delays. This might involve mandating risk management training for construction professionals, promoting standardized risk assessment methodologies, establishing a central risk mitigation information hub, fostering better communication channels with local communities, establishing conflict resolution mechanisms, and promoting community engagement initiatives.

This comprehensive analysis underscores the critical role of security in Afghan construction delays while acknowledging the multifaceted nature of the challenge and the diverse perspectives of stakeholders. By proposing targeted interventions, advocating for a holistic approach to risk management, highlighting avenues for future research, and outlining potential policy recommendations, this study offers a valuable roadmap for enhancing project resilience and fostering a more sustainable construction sector in Afghanistan and beyond.

### 6.2 Recommendations

Stakeholders should prioritize developing and implementing robust security protocols, including measures to enhance site security, foster community engagement, and improve coordination with local authorities and security forces to ensure timely responses to threats. Adopting a proactive approach to risk management that integrates security considerations into project planning and decision-making is crucial, including conducting comprehensive risk assessments, developing contingency plans, and investing in resilience-building measures like supply chain diversification and remote monitoring technologies. Further studies should explore the effectiveness of such interventions. This research provides valuable insights for developing evidence-based policies and practices promoting sustainable development in conflict-affected regions.

## Data availability statement

The data used in this study are available upon request.

## CRediT authorship contribution statement

**Mohammad Basheer Ahmadzai:** Writing – review & editing, Writing – original draft, Visualization, Validation, Software, Resources, Methodology, Investigation, Formal analysis, Data curation, Conceptualization. **Kunhui Ye:** Writing – review & editing, Validation, Supervision, Project administration, Methodology, Funding acquisition, Data curation, Conceptualization.

## Declaration of competing interest

The authors declare that they have no known competing financial interests or personal relationships that could have appeared to influence the work reported in this paper.
